# New and confirmed records of fruit flies (Diptera, Tephritidae) from Italy

**DOI:** 10.3897/BDJ.9.e69351

**Published:** 2021-08-31

**Authors:** Luca Mazzon, Daniel Whitmore, Pierfilippo Cerretti, Valery A. Korneyev

**Affiliations:** 1 Department of Agronomy, Food, Natural Resources, Animals and Environment (DAFNAE), University of Padua, Padua, Italy Department of Agronomy, Food, Natural Resources, Animals and Environment (DAFNAE), University of Padua Padua Italy; 2 Staatliches Museum für Naturkunde Stuttgart, Stuttgart, Germany Staatliches Museum für Naturkunde Stuttgart Stuttgart Germany; 3 Department of Biology and Biotechnology 'Charles Darwin', Sapienza University of Rome, Rome, Italy Department of Biology and Biotechnology 'Charles Darwin', Sapienza University of Rome Rome Italy; 4 I.I.Schmalhausen Institute of Zoology, Kyiv, Ukraine I.I.Schmalhausen Institute of Zoology Kyiv Ukraine

**Keywords:** Diptera, Tephritidae, Italy, checklist, additions

## Abstract

**Background:**

Prior to this study, 141 species of Tephritidae were known to occur in Italy.

**New information:**

Italian records of nine species of the family Tephritidae (Diptera) are provided. Five species, *Eurasimonastigma* (Loew, 1840), *Noeetabisetosa* Merz, 1992, *Campiglossadoronici* (Loew, 1856), *Xyphosialaticauda* (Meigen, 1826) and *Rhagoletisberberidis* Jermy, 1961 are recorded from Italy for the first time, whereas four species, *Inuromaesamaura* (Frauenfeld, 1857), *Urophoracuspidata* (Meigen, 1826), *Tephritisconyzifoliae* Merz, 1992 and *T.mutabilis* Merz, 1992, previously recorded in the Fauna Europaea database without reference to collection material, are confirmed and supplemented with host plant data and other collection data.

## Introduction

Tephritidae is one of the largest families of cyclorrhaphous Diptera, with almost 5,000 named species predominantly found in the tropics (A. Norrbom, pers. comm.). The family is also referred to as “fruit flies”, as it contains many species that are specialised feeders on fleshy fruit. The tropical regions are true hotspots of tephritid diversity, including hundreds of fruit-eating species, but also species with saprophagous larvae feeding under the bark of fallen trees or in bamboo culms (Korneyev 1999, Dohm et al. 2014). On the other hand, most Palaearctic species are flower and seed feeders, with larvae that are borers in the flower heads, stems and rhizomes of Asteraceae, Lamiaceae and Acanthaceae. The European fauna is represented by at least 265 species belonging to three subfamilies: Dacinae, Tephritinae and Trypetinae (Merz and Korneyev 2011, V. Korneyev, unpublished data). Some of the fruit-eating species are widespread economic pests which cause serious damage to fruit crops and stored fruit. The olive fly, *Bactroceraoleae* (Rossi, 1794) and the medfly, *Ceratitiscapitata* (Wiedemann, 1817), as well as many members of the genera *Rhagoletis* Loew, 1862 and *Carpomya* Costa, 1854, are well-known examples.

Starting from Rondani (Rondani 1856, Rondani 1870), who listed 109 nominal species known from Italy and following numerous nomenclatural changes, the number of Italian fruit fly species reached 133 species according to Belcari et al. (1995). Later, Merz and Korneyev (2011) increased this number to 141 species, but did not provide occurrence data for species added to the Italian list. These were: *Inuromaesamaura* (Frauenfeld, 1857), *Urophoracongrua* Loew, 1862, *U.cuspidata* (Meigen, 1826), *Tephritisconyzifoliae* Merz, 1992, *T.maccus* Hering, 1937, *T.mutabilis* Merz, 1992, *Chaetorelliaacrolophi* White & Marquardt, 1989, Terellia (Cerajocera) rhapontici Merz, 1990, *Rhagoletisbatava* Hering, 1958 and *R.cingulata* (Loew, 1862), all based on collection material identified by Bernhard Merz, but not formally published.

Other relatively recent contributions to the Italian tephritid fauna, including first Italian records, were made by Merz (2002) for *Campiglossamisella* (Loew, 1869) and Rivosecchi (2008) for *Euphrantatoxoneura* (Loew, 1846). Gentilini et al. (2006) described two fossil species from the Upper Miocene (Messinian) of Monte Castellaro. Seljak (2013) recorded *Euarestaaequalis* (Loew), a non-native, North American species considered beneficial as a biological control agent of the invasive plant *Xanthiumstrumarium* L. (Asteraceae), from Italy and Slovenia for the first time. Recently, Nugnes et al. (2018) provided the first Italian records of the invasive fruit pest species *Bactroceradorsalis* (Hendel, 1912) from the Region of Campania.

In this paper, we record five tephritid species for Italy for the first time and confirm the occurrence in the country of four additional species, based on detailed collection data and host plant information obtained during research on symbiotic bacteria of over 30 fruit fly species. The analysis revealed the presence of hereditary symbiotic bacteria in *Bactroceraoleae* (Rossi, 1790) (Capuzzo et al. 2005) and in all studied species of the tribe Tephritini and the genus *Noeeta* Robineau-Desvoidy, 1830. In other tribes (e.g., Myopitini, Xyphosiini and Terelliini), despite the common trait of larvae developing in Asteraceae flower heads, evolution does not seem to have occurred for the establishment of a hereditary bacterial interaction (Mazzon et al. 2008, Mazzon et al. 2010, Mazzon et al. 2011).

## Materials and methods

The flies were collected in Italy by the first author, reared from mature larvae and pupae collected together with infested flower heads. Flower heads were detached from their stems and placed in net bags at room temperature to allow the insects to complete their development. An in-field pre-screening of positive samples involved sectioning of the inflorescences and inspection for the presence of larvae or pupae. Adults of *Eurasimonastigma* (Loew, 1840) were collected with a mouth aspirator while resting on their host plant.

The species were identified using the keys of White (1988) and Merz (1994). The nomenclature follows Norrbom et al. (1999). Host plants were determined using the Italian botanical keys in Pignatti (1982); when necessary, identifications were confirmed by a specialist.

All voucher specimens (Figs 1, 2) are deposited in the fruit fly collection of the Laboratory of Entomology of the Department of Agronomy, Food, Natural Resources, Animals and Environment, University of Padua, Italy (UPI).

## Taxon treatments

### Eurasimona stigma

(Loew, 1840)

F0AC0B9E-DFDA-5970-A4FC-7A485828C29F

#### Materials

- **Type status:**Other material. **Occurrence:** recordedBy: L. Mazzon; individualCount: 3; sex: male; lifeStage: adult; preparations: dry; **Taxon:** scientificName: *Eurasimonastigma* (Loew, 1840); higherClassification: Subfamily Tephritinae, Tribe Myopitini; genus: Eurasimona; specificEpithet: *stigma*; scientificNameAuthorship: (Loew, 1840); **Location:** continent: Europe; country: Italy; countryCode: I; stateProvince: Veneto Region; county: Padova Province; locality: Euganean Hills; verbatimElevation: 250 m; verbatimCoordinates: 45°19'16.08"N 11°42'20.06"E; decimalLatitude: 45.3211; decimalLongitude: 11.7055; georeferenceSources: Google Maps; **Identification:** identifiedBy: L. Mazzon; dateIdentified: 2006; **Event:** samplingProtocol: mouth aspirator; eventDate: 14/06/2006; habitat: edge of forest, on *Achillea* flowers; **Record Level:** basisOfRecord: PreservedSpecimen

#### Distribution

Austria, Czechia, Estonia, Germany, Finland, France, Hungary, Latvia, Lithuania, North Macedonia, Moldova, Slovakia, Sweden, Ukraine (Korneyev and White 1991, Merz and Korneyev 2011); Russia, east to southern Siberia (Krasnoyarsk); Kazakhstan; Turkmenistan; Uzbekistan; Kyrghyzstan (Korneyev and White 1991); Iran (Zarghani et al. 2016). The species (Fig. 1a) is here recorded as **new to Italy**.

#### Biology

The biology of *E.stigma* is poorly known. The flies are said to have been reared from *Leucanthemumvulgare* Lam. (Roser 1840, as “*Chrysanthemumleucanthemum*”), *Achilleamillefolium* L. (Loew 1862: 68), *Anthemisarvensis* L. (Korneyev et al. 2005; forming small non-lignified galls in a single flower head), *A.cotula* L. (Hendel 1927) and *Tanacetumvulgare* L. (Merz 1994), but there are no comprehensive rearing data or descriptions of its biology.

### Inuromaesa maura

(Frauenfeld, 1857)

5DD226B4-8501-55DD-8824-A3AC72E27A17

#### Materials

- **Type status:**Other material. **Occurrence:** recordedBy: L. Mazzon; individualCount: 5; sex: 4 males and 1 female; lifeStage: adult, reared from immature stages; preparations: dry whole insect; **Taxon:** scientificName: *Inuromaesamaura* (Frauenfeld, 1857); higherClassification: Subfamily Tephritinae, Tribe Myopitini; genus: Inuromaesa; specificEpithet: *maura*; scientificNameAuthorship: (Frauenfeld, 1857); **Location:** continent: Europe; country: Italy; countryCode: I; stateProvince: Friuli-Venezia Giulia Region; county: Pordenone Province; locality: Montereale Val Cellina; verbatimElevation: 1100 m; verbatimCoordinates: 46°10'00.0"N 12°36'00.0"E; decimalLatitude: 46.1668; decimalLongitude: 12.6000; georeferenceSources: Google Maps; **Identification:** identifiedBy: L. Mazzon; dateIdentified: 2005; **Event:** samplingProtocol: from flower heads of *Inulahirta*; eventDate: 25/06/2005; habitat: edge of forest; **Record Level:** basisOfRecord: PreservedSpecimen

#### Distribution

Austria, Czechia, central and southern France, Hungary, Italy, Slovakia, northern Spain, Switzerland and Ukraine (Merz and Korneyev 2011); Russia: West Siberia; Kazakhstan (Korneyev and White 1999); Iran (Zarghani et al. 2016). Note: the present records from Italy (Fig. 1b) confirm the country-level record by Merz and Korneyev (2011).

#### Biology

The larvae develop in the achenes of *Pentanemahirtum* (L.) D.Gut.Larr. et al. (= *Inulahirta*), *Pentanemaoculus-christi* (L.) D.Gut.Larr. et al. (= *Inulaoculus-christi*) (Frauenfeld 1857), *Pentanemamontanum* (L.) D.Gut.Larr. et al. (= *Inulamontana*) (Anonymous 1934), *Pentanemaensifolium* (L.) D.Gut.Larr. et al. (= *Inulaensifolia*) (Mihalyi 1960, Korneyev and White 1991) and *Pentanemasalicinum* (L.) D.Gut.Larr. et al. (= *Inulasalicina*) (Richter 1988), forming no obvious galls.

### Urophora cuspidata

(Meigen, 1826)

6E9FDE32-3C4D-5944-A7B0-57DE0C2794FA

#### Materials

- **Type status:**Other material. **Occurrence:** recordedBy: V. Girolami; individualCount: 2; sex: 1 male and 1 female; lifeStage: adult; preparations: dry whole insect; **Taxon:** scientificName: *Urophoracuspidata* (Meigen, 1826); higherClassification: Subfamily Tephritinae, Tribe Myopitini; genus: Urophora; specificEpithet: *cuspidata*; scientificNameAuthorship: (Meigen, 1826); **Location:** continent: Europe; country: Italy; countryCode: I; stateProvince: Friuli-Venezia Giulia Region; county: Pordenone Province; municipality: Fanna; locality: Via Vals; verbatimElevation: 320 m; verbatimCoordinates: 46°11'6.43"N 12°43'55.33"E; decimalLatitude: 46.1851; decimalLongitude: 12.7320; georeferenceSources: Google Maps; **Identification:** dateIdentified: 2007; **Event:** samplingProtocol: reared from flower heads of *Centaureascabiosa*; eventDate: 15/07/2006; habitat: grassland; **Record Level:** basisOfRecord: PreservedSpecimen
- **Type status:**Other material. **Occurrence:** recordedBy: V. Girolami; individualCount: 2; sex: 2 females; preparations: dry whole insect; **Taxon:** scientificName: *Urophoracuspidata* (Meigen, 1826); higherClassification: Subfamily Tephritinae, Tribe Myopitini; genus: Urophora; specificEpithet: *cuspidata*; scientificNameAuthorship: (Meigen, 1826); **Location:** continent: Europe; country: Italy; countryCode: I; stateProvince: Friuli-Venezia Giulia Region; county: Pordenone Province; municipality: Fanna; locality: Via Vals; verbatimElevation: 320 m; verbatimCoordinates: 46°11'6.43"N 12°43'55.33"E; decimalLatitude: 46.1851; decimalLongitude: 12.7320; georeferenceSources: Google Maps; **Identification:** dateIdentified: 2007; **Event:** samplingProtocol: reared from flower heads of *Centaureascabiosa*; eventDate: 26/06/2007; habitat: grassland; **Record Level:** basisOfRecord: PreservedSpecimen

#### Distribution

Northern, central and eastern Europe from northern Spain, southern France and Italy (Merz 1994, Merz and Korneyev 2011) to Ukraine, European and Asian Russia and Kazakhstan (Korneyev and White 1996); Iran (Mohamadzade Namin and Nozari 2011). Notes: the present records from Italy (Fig. 1c) confirm the country-level record by Merz and Korneyev (2011). The record from Turkey by Kütük et al. (2013) needs confirmation.

#### Biology

The larvae develop in flower heads of *Centaureascabiosa* L. (incl. ssp. alpestris) and *Ce.collina* L. (White and Korneyev 1989); records of other host species, including “*Ce.tenuifolia*" (Merz 1994) need confirmation.

### Noeeta bisetosa

Merz, 1992

DFFD5088-752B-5822-B430-0AACC79F4621

#### Materials

- **Type status:**Other material. **Occurrence:** recordedBy: V. Girolami; individualCount: 16; sex: 9 males and 7 females; lifeStage: adult; preparations: dry whole insect; **Taxon:** scientificName: *Noeetabisetosa* Merz, 1992; higherClassification: Subfamily Tephritinae, Tribe Noeetini; genus: Noeeta; specificEpithet: *bisetosa*; scientificNameAuthorship: Merz, 1992; **Location:** continent: Europe; country: Italy; countryCode: I; stateProvince: Friuli-Venezia Giulia Region; county: Pordenone Province; municipality: Fanna; locality: Val Cellina; verbatimElevation: 290 m; verbatimCoordinates: 46°10'11.8"N 12°40'02.7"E; decimalLatitude: 46.1699; decimalLongitude: 12.6674; georeferenceSources: Google Maps; **Identification:** identifiedBy: L. Mazzon; dateIdentified: 2006; **Event:** samplingProtocol: reared from flower heads of *Hieraciumpiloselloides*; eventDate: 10/07/2006; habitat: gravel streambed; **Record Level:** basisOfRecord: PreservedSpecimen

#### Distribution

Austria (Merz and Kofler 2008), Hungary (Merz 2000), Russia (Basov 1999), Switzerland (Merz 1992) and Ukraine (Korneyev 2003). The species (Fig. 2a) is here recorded as **new to Italy**.

#### Biology

The larvae feed in flower heads of *Hieraciumpiloselloides* Vill. (Merz 1992).

### Campiglossa doronici

(Loew, 1856)

9E43CCD2-F403-51B2-8FB5-C756DC4A93A9

#### Materials

- **Type status:**Other material. **Occurrence:** recordedBy: L. Mazzon; individualCount: 20; sex: 11 males and 9 females; lifeStage: adult; preparations: dry whole insect; **Taxon:** scientificName: *Campiglossadoronici* (Loew, 1856); higherClassification: Subfamily Tephritinae, Tribe Tephritini; genus: Campiglossa; specificEpithet: *doronici*; scientificNameAuthorship: (Loew, 1856); **Location:** continent: Europe; country: Italy; countryCode: I; stateProvince: Veneto Region; county: Vicenza Province; locality: Monte Cengio; verbatimElevation: 1320 m; verbatimCoordinates: 45°48'40.10"N 11°23'39.36"E; decimalLatitude: 45.8111; decimalLongitude: 11.3943; georeferenceSources: Google Maps; **Identification:** identifiedBy: L. Mazzon; dateIdentified: 2005; **Event:** samplingProtocol: reared from flower heads of *Doronicumaustriacum*; eventDate: 02/07/2005; habitat: edge of forest; **Record Level:** basisOfRecord: PreservedSpecimen

#### Distribution

Austria, Czechia, France, Poland, Romania, Slovakia and Ukraine (Merz and Korneyev 2011). The species (Fig. 2b) is here recorded as **new to Italy**.

#### Biology

The larvae feed in flower heads of *Doronicumaustriacum* Jacq. (Loew 1856).

### Tephritis conyzifoliae

Merz, 1992

8BFC5E9D-2525-57A3-A97E-C5DF4F52896A

#### Materials

- **Type status:**Other material. **Occurrence:** recordedBy: L. Mazzon; individualCount: 17; sex: 8 males and 9 females; lifeStage: adult; preparations: dry whole insect; **Taxon:** scientificName: *Tephritisconyzifoliae* Merz, 1992; higherClassification: Subfamily Tephritinae, Tribe Tephritini; genus: Tephritis; specificEpithet: *conyzifoliae*; scientificNameAuthorship: Merz, 1992; **Location:** continent: Europe; country: Italy; countryCode: I; stateProvince: Trentino-Alto Adige Region; county: Trento Province; municipality: Moena; locality: San Pellegrino Pass; verbatimElevation: 1925 m; verbatimCoordinates: 46°22'48.0"N 11°47'37.0"E; decimalLatitude: 46.3800; decimalLongitude: 11.7936; georeferenceSources: Google Maps; **Identification:** identifiedBy: L. Mazzon; dateIdentified: 2008; **Event:** samplingProtocol: reared from flower heads of *Crepisconyzifolia*; eventDate: 28/07/2008; habitat: pasture; **Record Level:** basisOfRecord: PreservedSpecimen

#### Distribution

Armenia (Evstigneev and Glukhova 2020), Czechia, France, Italy, Switzerland (Merz and Korneyev 2011), Russia (Evstigneev 2016), Kazakhstan, Kyrgyzstan (Korneyev 2016a), Poland (Klasa and Palaczyk 2005), Tajikistan (Korneyev and Korneyev 2019) and Ukraine (Korneyev and Klasa 2016). Note: the present records from Italy (Fig. 2c) confirm the country-level record by Merz and Korneyev (2011).

#### Biology

The larvae develop in flower heads of *Crepisconyzifolia* (Gouan) A. Kern. (Merz 1992), *Cr.sibirica* L. (Shcherbakov 2001, Korneyev 2016a),*Cr.pannonica* (Jacq.) K. Koch (Evstigneev 2016) and *Cr.ciliata* K. Koch (Evstigneev and Glukhova 2020).

#### Notes

This species was recorded from continental Italy by Merz and Korneyev (2011), without further collection data. Korneyev (2016a) treated *T.conyzifoliae* as a senior synonym of *Tephritisacademica* Bassov and Tolstoguzova, 1994, *T.nartshukovi* Bassov and Tolstoguzova, 1994 and *T.epicrepis* Scherbakov, 2001, all described from Russia.

### Tephritis mutabilis

Merz, 1992

E7BA4CEE-2710-5E79-A6E5-53139E8FBBE2

#### Materials

- **Type status:**Other material. **Occurrence:** recordedBy: V. Girolami; individualCount: 3; sex: 1 males and 2 females; lifeStage: adult; preparations: dry whole insect; **Taxon:** scientificName: *Tephritismutabilis* Merz, 1992; higherClassification: Subfamily Tephritinae, Tribe Tephritini; genus: Tephritis; specificEpithet: *mutabilis*; scientificNameAuthorship: Merz, 1992; **Location:** continent: Europe; country: Italy; countryCode: I; stateProvince: Friuli-Venezia Giulia Region; county: Pordenone Province; municipality: Fanna; verbatimElevation: 280 m; verbatimCoordinates: 46°11'12.3"N 12°44'39.7"E; decimalLatitude: 46.1867; decimalLongitude: 12.7444; georeferenceSources: Google Maps; **Identification:** identifiedBy: L. Mazzon; dateIdentified: 2007; **Event:** samplingProtocol: reared from flower heads of *Leontodonhispidus*; eventDate: 28/05/2007; habitat: grassland; **Record Level:** basisOfRecord: PreservedSpecimen

#### Distribution

Central Europe (Austria, Czechia, France, Germany, Italy, Poland, Slovakia, Switzerland) (Merz and Korneyev 2011), Finland (Kahanpää and Winqvist 2014), Russia (Korneyev 2016b) and Ukraine (Korneyev and Klasa 2016). Note: the present records from Italy (Fig. 2d) confirm the country-level record by Merz and Korneyev 2011.

#### Biology

The larvae feed in flower heads of *Leontodonhispidus* L. (Merz 1992, Merz 1994).

### Xyphosia laticauda

(Meigen, 1826)

A311316E-33A9-5F21-84B5-8B731A9CAC09

#### Materials

- **Type status:**Other material. **Occurrence:** recordedBy: V. Girolami; individualCount: 1; sex: male; lifeStage: adult; preparations: dry whole insect; **Taxon:** scientificName: *Xyphosialaticauda* (Meigen, 1826); higherClassification: Subfamily Tephritinae, Tribe Xyphosiini; genus: Xyphosia; specificEpithet: *laticauda*; scientificNameAuthorship: (Meigen, 1826); **Location:** continent: Europe; country: Italy; countryCode: I; stateProvince: Friuli-Venezia Giulia Region; county: Pordenone Province; municipality: Maniago; locality: Colvera; verbatimElevation: 210 m; verbatimCoordinates: 46°10'11.11"N, 12°44'16.48"E; decimalLatitude: 46.1698; decimalLongitude: 12.7379; georeferenceSources: Google Maps; **Identification:** identifiedBy: L. Mazzon; dateIdentified: 2006; **Event:** samplingProtocol: reared from flower heads of *Centaureatriumfettii*; eventDate: 10/07/2006; habitat: gravel streambed; **Record Level:** basisOfRecord: PreservedSpecimen
- **Type status:**Other material. **Occurrence:** recordedBy: V. Girolami; individualCount: 1; sex: male; lifeStage: adult; preparations: dry whole insect; **Taxon:** scientificName: *Xyphosialaticauda* (Meigen, 1826); higherClassification: Subfamily Tephritinae, Tribe Xyphosiini; genus: Xyphosia; specificEpithet: *laticauda*; scientificNameAuthorship: (Meigen, 1826); **Location:** continent: Europe; country: Italy; countryCode: I; stateProvince: Friuli-Venezia Giulia Region; county: Pordenone Province; municipality: Cavasso Nuovo; locality: Meduna; verbatimElevation: 147 m; verbatimCoordinates: 46°12'41.0"N 12°46'38.9"E; decimalLatitude: 46.2114; decimalLongitude: 12.7775; georeferenceSources: Google Maps; **Identification:** identifiedBy: L. Mazzon; dateIdentified: 2006; **Event:** samplingProtocol: reared from flower heads of *Centaureatriumfettii*; eventDate: 07/07/2006; habitat: gravel streambed; **Record Level:** basisOfRecord: PreservedSpecimen

#### Distribution

Austria, France, Hungary, Switzerland (Merz and Korneyev 2011); Armenia (Korneyev 1983); Russian North Caucasus (Korneyev and Korneyev, unpublished data). The species (Fig. 2e) is here recorded as **new to Italy**.

#### Biology

The larvae develop in flower heads of *Centaureamontana* L. (Frauenfeld 1857); this species is superficially similar to *Centaureatriumfettii* All.; either both species are infested by this species or misidentifications have occurred.

### Rhagoletis berberidis

Jermy, 1961

A42F684A-1BBC-569D-A4CA-C07EDE92372C

#### Materials

- **Type status:**Other material. **Occurrence:** recordedBy: V. Girolami; individualCount: 2; sex: males; lifeStage: adult; preparations: dry whole insect; **Taxon:** scientificName: *Rhagoletisberberidis* Jermy, 1961; higherClassification: Subfamily Trypetinae, Tribe Carpomyini; genus: Rhagoletis; specificEpithet: *berberidis*; scientificNameAuthorship: Jermy, 1961; **Location:** continent: Europe; country: Italy; countryCode: I; stateProvince: Friuli-Venezia Giulia Region; county: Pordenone Province; municipality: Claut; verbatimElevation: 675 m; verbatimCoordinates: 46°16'6.14"N, 12°31'39.23"E; decimalLatitude: 46.2684; decimalLongitude: 12.5276; georeferenceSources: Google Maps; **Identification:** identifiedBy: L. Mazzon; dateIdentified: 2009; **Event:** samplingProtocol: reared from fruits of *Berberisvulgaris*; eventDate: 15/08/2008; habitat: edge of forest; **Record Level:** basisOfRecord: PreservedSpecimen

#### Distribution

Austria, Hungary, Slovakia, Switzerland, Ukraine (Merz and Korneyev 2011); Russian North Caucasus (Kandybina 1977), Turkey (Kütük and Özaslan 2006) and Iran (Mohamadzade Namin et al. 2010). The species (Fig. 2f) is here recorded as **new to Italy**.

#### Biology

The larvae develop in seeds of *Berberisvulgaris* L. (Jermy 1961, Merz 1994).

## Discussion

Based on the present results and on a recent revision of the fauna, the revised checklist of Italian Tephritidae (Mazzon and Korneyev, in press) includes 151 extant and two fossil species.

## Supplementary Material

XML Treatment for Eurasimona stigma

XML Treatment for Inuromaesa maura

XML Treatment for Urophora cuspidata

XML Treatment for Noeeta bisetosa

XML Treatment for Campiglossa doronici

XML Treatment for Tephritis conyzifoliae

XML Treatment for Tephritis mutabilis

XML Treatment for Xyphosia laticauda

XML Treatment for Rhagoletis berberidis

## Figures and Tables

**Figure 1a.**
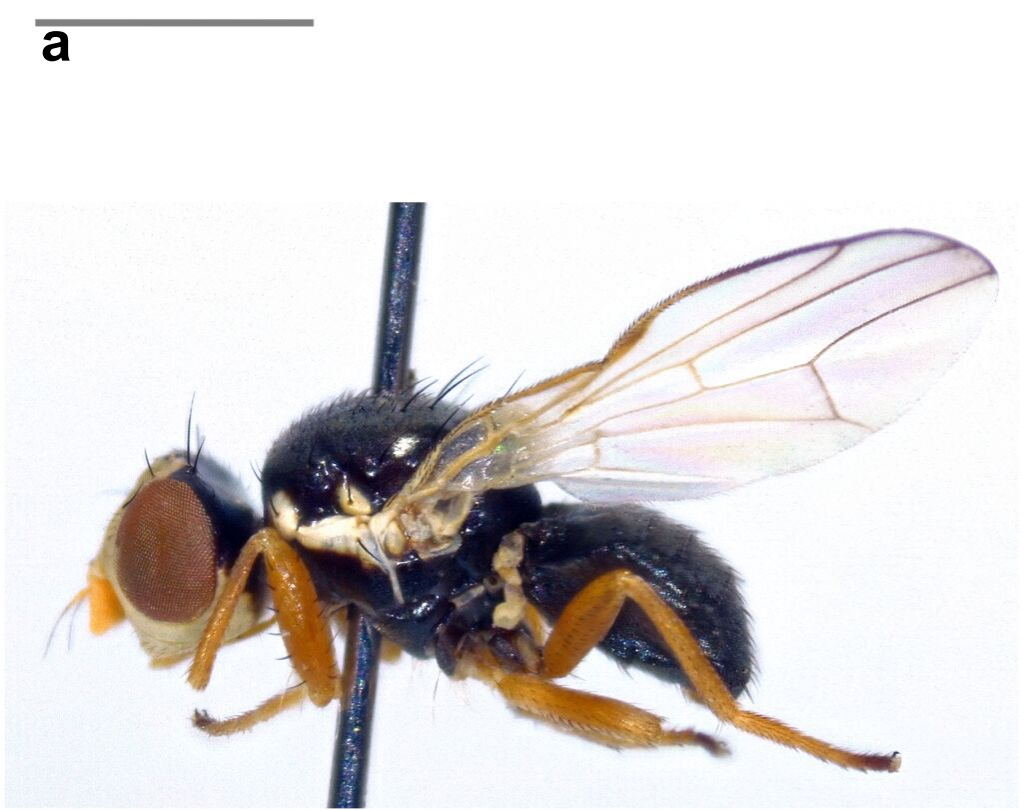
*Eurasimonastigma* (Loew, 1840)

**Figure 1b.**
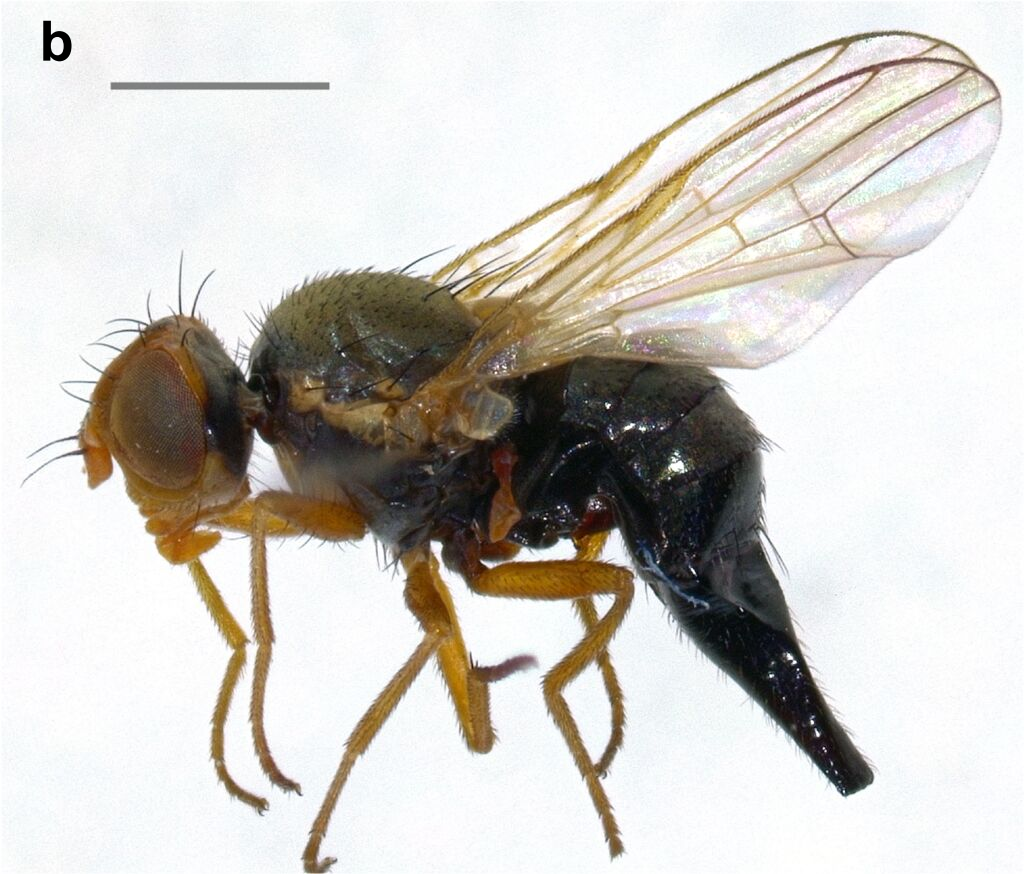
*Inuromaesamaura* (Frauenfeld, 1857)

**Figure 1c.**
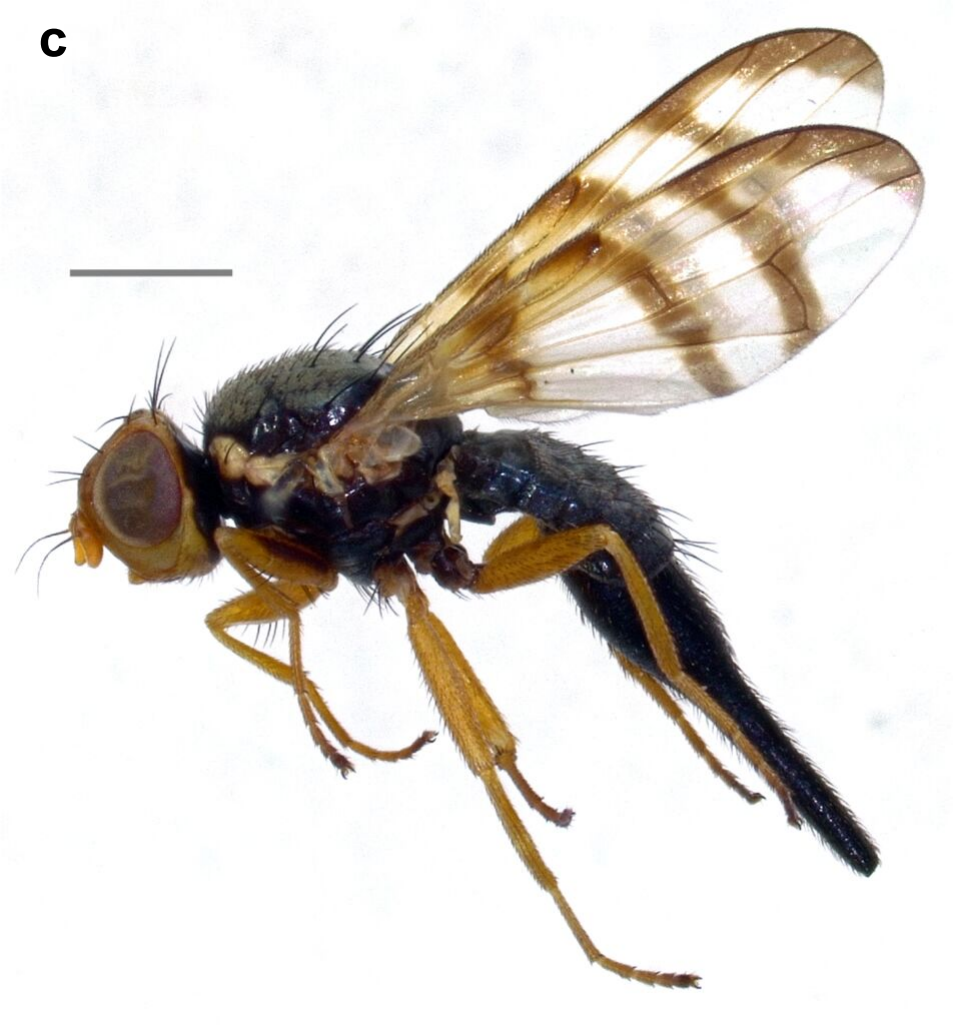
*Urophoracuspidata* (Meigen, 1826)

**Figure 2a.**
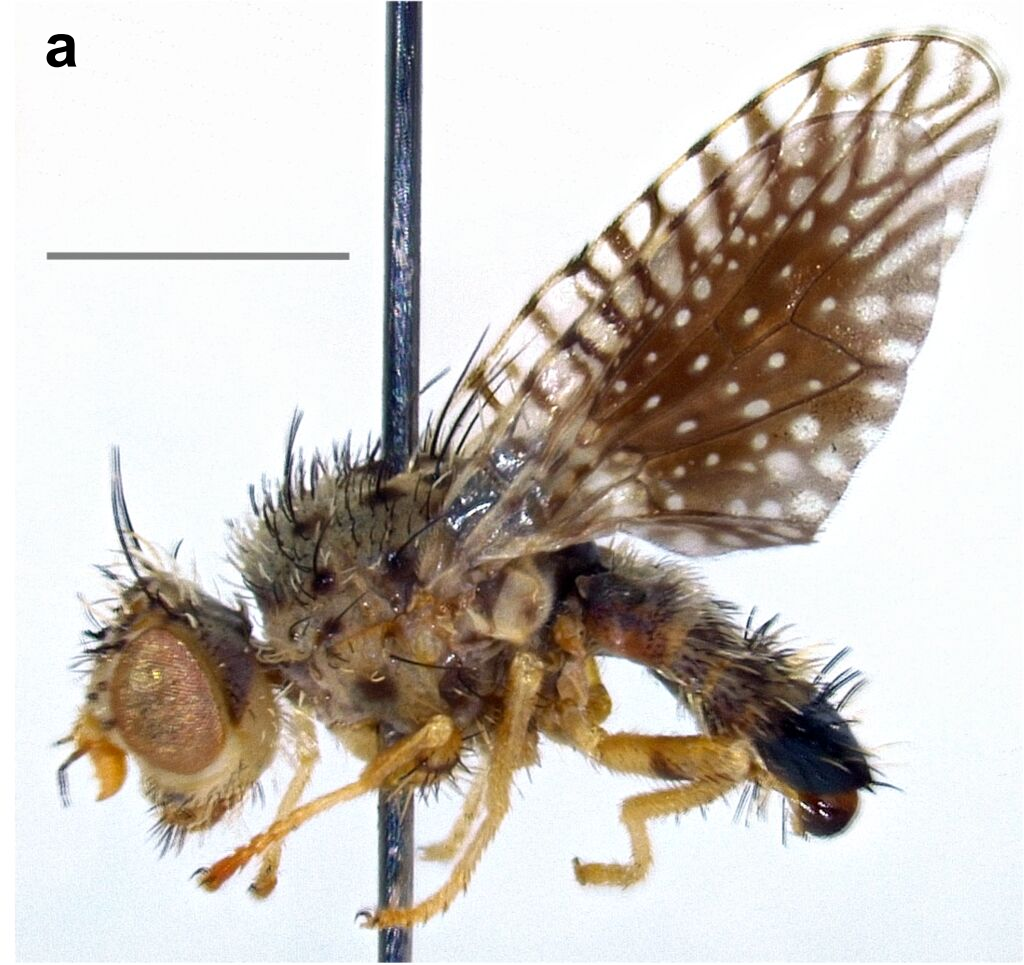
*Noeetabisetosa* Merz, 1992

**Figure 2b.**
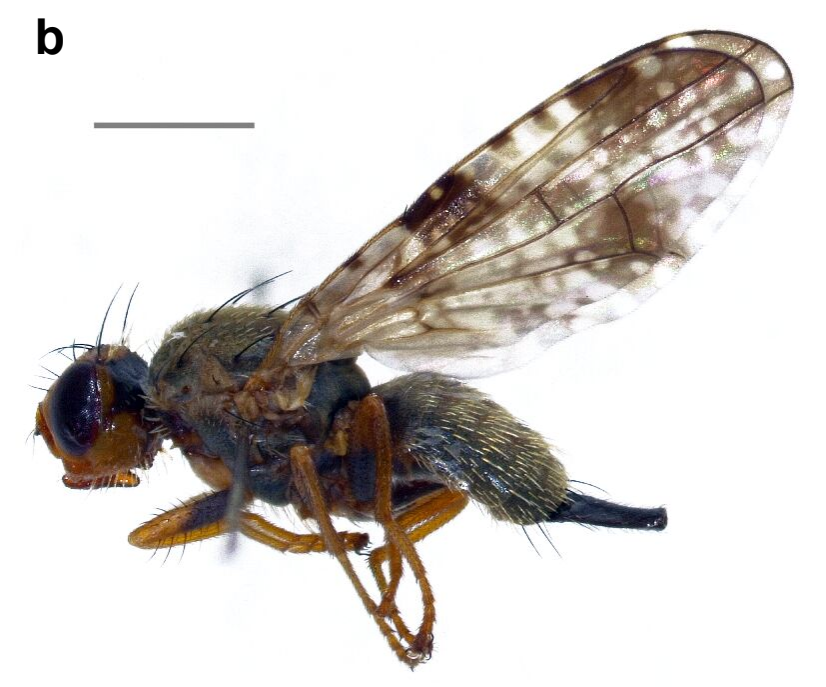
*Campiglossadoronici* (Loew, 1856)

**Figure 2c.**
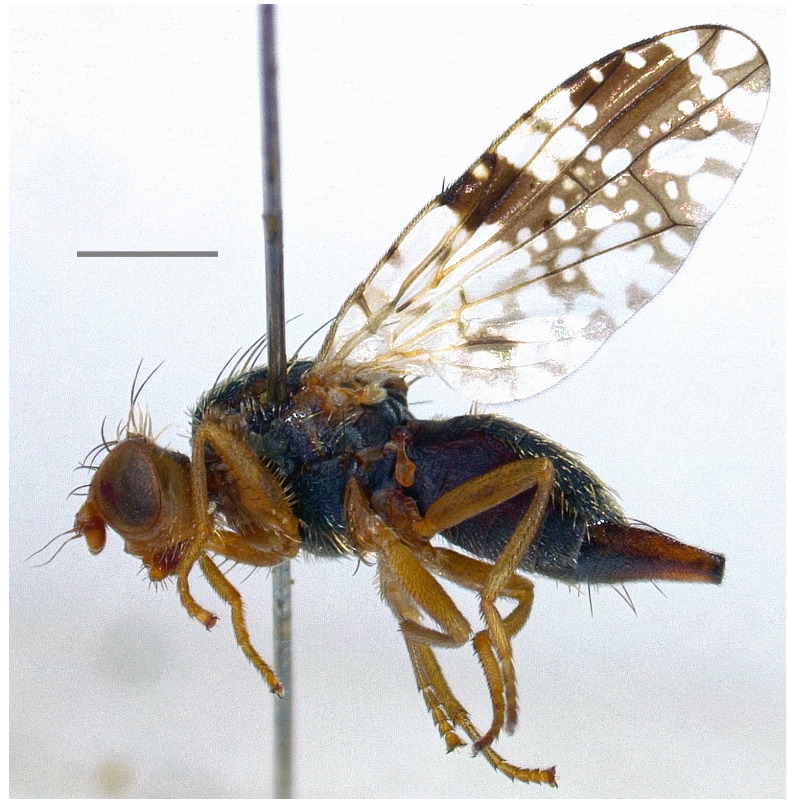
*Tephritisconyzifoliae* Merz, 1992

**Figure 2d.**
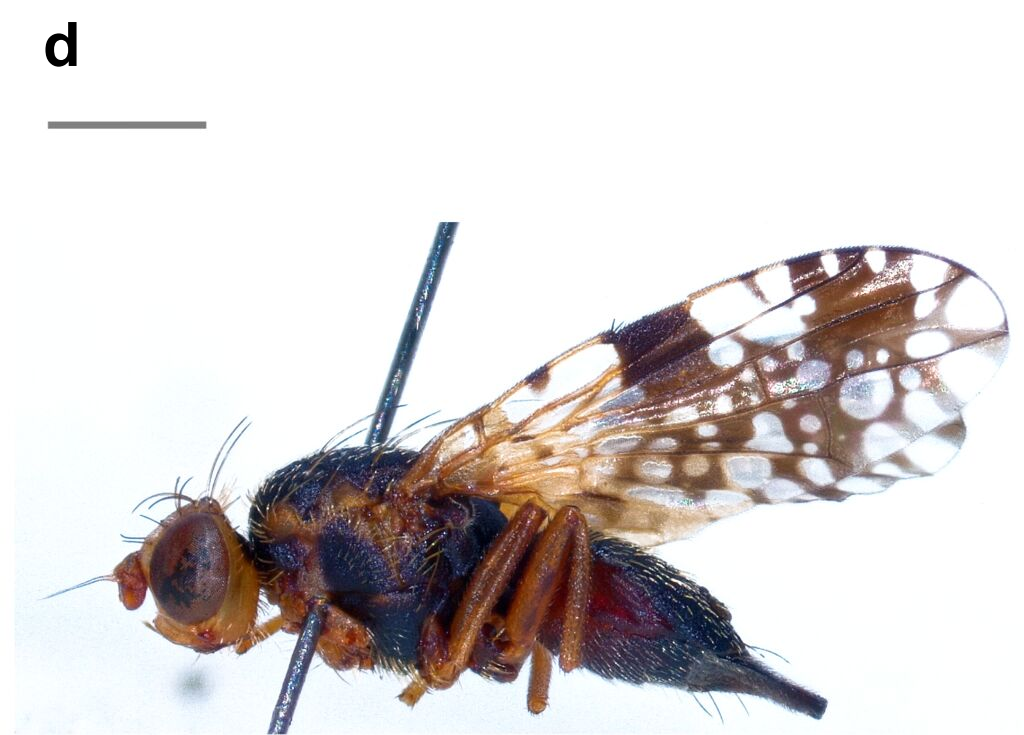
*Tephritismutabilis* Merz, 1992

**Figure 2e.**
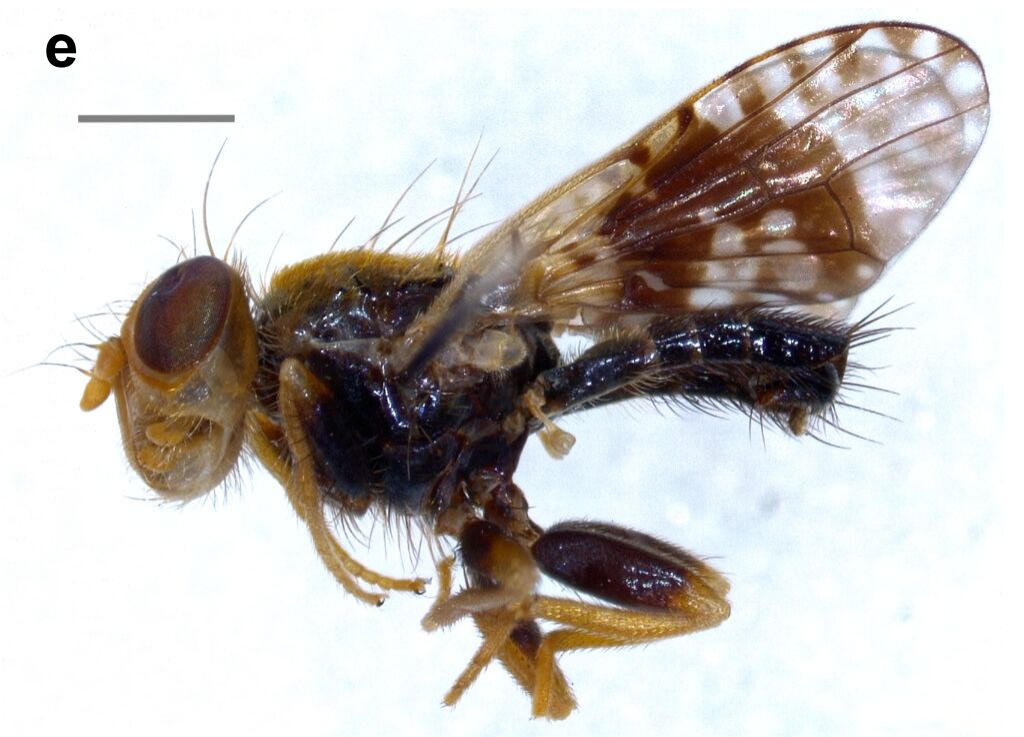
*Xyphosialaticauda* (Meigen, 1826)

**Figure 2f.**
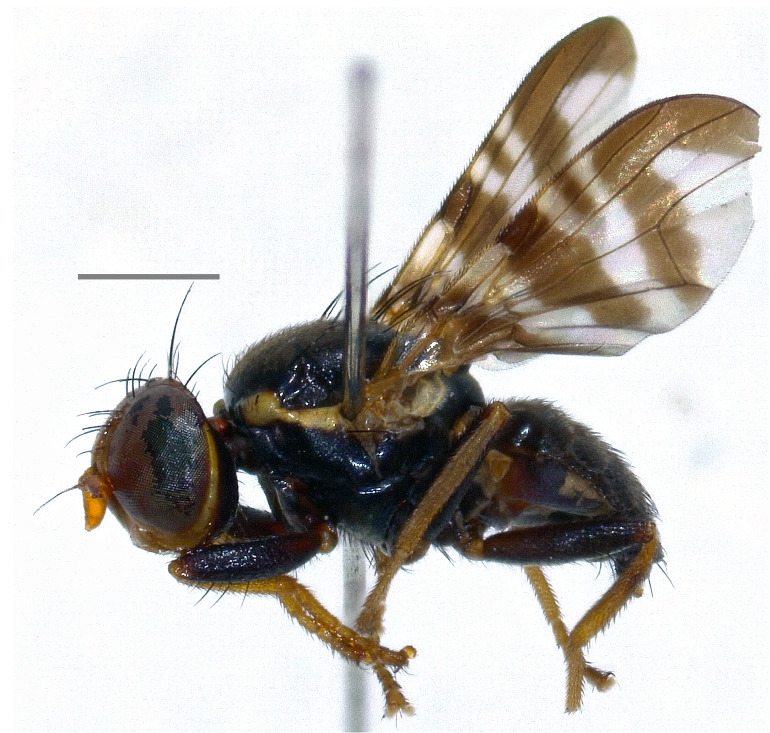
*Rhagoletisberberidis* Jermy, 1961

## References

[B7077813] Basov V. M. (1999). Tephritidae (Diptera) of Middle Volga and Cis-Ural areas. Vestnik Udmurtskogo Universiteta. Seria Biologicherskoe Raznoobrazie Udmurtskoy Republiki.

[B7077822] Belcari A., Girolami V., Rivosecchi L., Zaitzev V. F., Minelli A, Ruffo S, La Posta S (1995). Checklist delle specie della fauna italiana.

[B7133735] Capuzzo C., Firrao G., Mazzon L., Squartini A., Girolami V. (2005). '*Candidatus* Erwinia dacicola', a coevolved symbiotic bacterium of the olive fly *Bactroceraoleae* (Gmelin). International Journal of Systematic and Evolutionary Microbiology.

[B7077835] Dohm P., Kovac D., Freidberg A., Rull J., Aluja M. (2014). Basic biology and host use patterns of tephritid flies (Phytalmiinae: Acanthonevrini, Dacinae: Gastrozonini) breeding in bamboo (Poaceae: Bambusoideae). Annals of the Entomological Society of America.

[B7077854] Evstigneev D. A. (2016). Tephritid flies of the Higher Tephritines group (Diptera, Tephritidae, Tephritinae) of Ulyanovsk and Samara Regions (Russia). Ukrainska Entomofaunistyka.

[B7077845] Evstigneev D. A., Glukhova N. V. (2020). First records of two species of Tephritidae and one species of Platystomatidae (Diptera) from Transcaucasia. Zoosystematica Rossica.

[B7077890] Frauenfeld G. R. (1857). Beitrage zur Naturgeschichte der Trypeten nebst 1537 Beschreibung einiger neuer Arten.. Sitzungsberichte der Akademie der Wissenschaften in Wien.

[B7077872] Gentilini G., Korneyev V. A., Kameneva E. P. (2006). Fossil tephritoid flies (Diptera: Pallopteridae, Ulidiidae, Tephritidae) from the Upper Miocene of Monte Castellaro, Italy, and a review of fossil European tephritoids. Instrumentas Biodiversitatis, Geneva.

[B7081928] Hendel F., Lindner E. (1927). Die Fliegen der palaearktischen Region.

[B7077881] Jermy T. (1961). Eine neue *Rhagoletis*-Art (Diptera: Trypetidae) aus den Fruchten von *Berberisvulgaris* L. Acta Zoologica Hungarica.

[B7077900] Kahanpää J., Winqvist K. (2014). Checklist of the Diptera superfamilies Tephritoidea and Sciomyzoidea of Finland (Insecta). ZooKeys.

[B7077909] Kandybina M. N. (1977). Larvae of fruit-infesting fruit flies (Diptera, Tephritidae). Opredeliteli po Faune SSSR.

[B7077918] Klasa A., Palaczyk A. (2005). Nasionnicowate (Tephritidae, Diptera) polskich Karpat stan poznania. Biuletyn Muzeum Przyrodniczego w Krakowie.

[B7077927] Korneyev S. V. (2016). On the taxonomic revision of the genus *Tephritis* (Diptera, Tephritidae): new synonymy. Vestnik Zoologii.

[B7077936] Korneyev S. V. (2016). *Tephritismutabilis* Merz (Diptera: Tephritidae): first record from Asia. Ukrainska Entomofaunistyka.

[B7077954] Korneyev S. V., Klasa A. (2016). New records and a revised checklist of fruit flies of the genus *Tephritis* (Diptera, Tephritidae) from Ukraine. Vestnik Zoologii.

[B7077945] Korneyev S. V., Korneyev V. A. (2019). Revision of the Old-World species of the genus *Tephritis* (Diptera, Tephritidae) with a pair of isolated apical spots. Zootaxa.

[B7081919] Korneyev V. A. (1983). *Xyphosialaticauda* Mg., a species of tephritid flies (Diptera: Tephritidae) new for the USSR fauna. Vestnik zoologii.

[B7077985] Korneyev V. A., White I. M. (1991). Fruit flies of the genus *Urophora* R.-D. (Diptera, Tephritidae) of the East Palearctic. I. A key to subgenera and review of species (except the subgenus Urophora s. str. Entomological Review (Washington.

[B7077994] Korneyev V. A., White I. M. (1996). Fruit-flies of the genus *Urophora* R.-D. (Diptera: Tephritidae) of Eastern Palaearctics. II. Review of species of the subgenus Urophora s. str. Communication 3. Entomological Review (Washington).

[B7077963] Korneyev V. A., Aluja M., Norrbom A. L. (1999). Fruit flies (Tephritidae): Phylogeny and evolution of behavior.

[B7081955] Korneyev V. A., White I. M. (1999). Tephritidae of the genus *Urophora* R.-D. (Diptera, Tephritidae of east palaearctic: III. Key to palaearctic species. Entomological Review.

[B7077976] Korneyev V. A. (2003). New and little-known Tephritidae (Diptera, Cyclorrhapha) from Europe. Vestnik Zoologii.

[B7078011] Korneyev V. A., Zwölfer H., Seitz A., Raman A., Schaefer C. W., Withers T. M. (2005). Biology, ecology and evolution of gall-inducing arthropods.

[B7078024] Kütük M., Özaslan M, (2006). Faunistical and systematical studies on the Trypetinae (Diptera: Tephritidae) in the Turkey along with a new record to Turkish fauna. Munis Entomology and Zoology.

[B7078033] Kütük M., Yaran M., Hayat R., Koyuncu M., Görmez V., Aytekin H. U. (2013). The determination of fruit fly (Diptera: Tephritidae) fauna in Adyaman, Kilis, and anlurfa provinces with a new record for Turkish fauna. Turkish Journal of Zoology.

[B7082004] Loew H. (1856). Neue Beitrage zur Kenntniss der Dipteren..

[B7078057] Loew H. (1862). Die europäische Bohrfliegen (Trypetidae).

[B7078065] Mazzon L., Piscedda A., Simonato M., Martinez-Sañudo I., Squartini A., Girolami V. (2008). Presence of specific symbiotic bacteria in flies of the subfamily Tephritinae (Diptera
Tephritidae) and their phylogenetic relationships: proposal of *Candidatus* Stammerula tephritidis. International Journal of Systematic and Evolutionary Microbiology.

[B7078076] Mazzon L., Martinez-Sañudo I., Simonato M., Squartini A., Savio C., Girolami V. (2010). Phylogenetic relationships between flies of the Tephritinae subfamily (Diptera, Tephritidae) and their symbiotic bacteria. Molecular Phylogenetics and Evolution.

[B7133605] Mazzon Luca, Martinez‑Sañudo Isabel, Savio Claudia, Simonato Mauro, Squartini Andrea, Zchori-Fein Einat, Bourtzis Kostas (2011). Manipulative tenants: bacteria associated with arthropods,.

[B7078087] Merz B. (1992). Fünf neue Fruchtfliegenarten aus den Schweizer Alpen und systematische Bemerkungen zu einigen europischen Gattungen und Arten (Diptera, Tephritidae). Mitteilungen der Schweizerischen Entomologischen Gesellschaft.

[B7078096] Merz B. (1994). Diptera: Tephritidae. Insecta Helvetica Fauna.

[B7078104] Merz B. (2000). Additions and corrections to the checklist of Tephritidae of Hungary (Diptera: Acalyptrata. Folia Entomologica Hungarica.

[B7078113] Merz B., Mason F, Cerretti P, Tagliapietra A, Speight MCD, Zapparoli M. (2002). Invertebrati di una foresta della Pianura Padana, Bosco della Fontana. Conservazione habitat Invertebrati 1..

[B7078127] Merz B., Kofler A. (2008). Fruchtfliegen aus Osttirol und Krnten (sterreich) (Diptera: Tephritidae). Linzer Biologische Beitrge.

[B7078136] Merz B, Korneyev VA Fauna Europaea: Tephritidae. Fauna Europaea version 2.4. https://fauna-eu.org.

[B7078145] Mihalyi F. (1960). Frlegyek. Trypetidae. Magyarorszg llatvilga.

[B7078163] Mohamadzade Namin S,, Nozari J,, Rasoulian Gh, (2010). The fruit flies (Diptera, Tephritidae) in Tehran Province, with new records for Iranian fauna. Vestnik Zoologii.

[B7078154] Mohamadzade Namin S., Nozari J. (2011). The fruit flies (Diptera: Tephritidae) in Kurdistan province, with new records for Iranian fauna. Ukrainska Entomofaunistyka.

[B7078172] Norrbom A. L., Carroll L. E., Thompson F. C., White I. M., A Freidberg, Thompson F. C. (1999). Fruit fly Expert Identification System and Systematic Information Database. Myia, 9..

[B7078186] Nugnes F., Russo E., Viggiani G., Bernardo U. (2018). First record of an invasive fruit fly belonging to Bactroceradorsalis complex (Diptera: Tephritidae) in Europe. Insects.

[B7133596] Pignatti S (1982). Flora d'Italia.

[B7078195] Richter V. A., Bei-Bienko G. Y. (1988). Keys to the insects of the European part of the USSR. Diptera and Siphonaptera. Part 2.

[B7321087] Rivosecchi L. (2008). Aggiunte e correzioni alle checklist di alcune famiglie di Ditteri della fauna italiana (Diptera). Bollettino della Società Entomologica Italiana.

[B7078217] Rondani C. (1856). *Dipterologiae Italicae prodromus. Vol. I: Genera Italica ordinis dipterorum ordinatim disposita et distincta et in familiae et stirpes aggregata*.. A. Stocchi, Parmae [= Parma],.

[B7078226] Rondani C. (1870). *Dipterologiae Italicae prodromus. Genera Italica ordinis dipterorum ordinatim disposita et distincta et in familias et stirpes aggregata*. 7 (pars 4) (sect. 1). A. Stocchi, Parmae [= Parma],.

[B7078235] Roser K. L.F. (1840). Erster Nachtrag zu dem im Jahre 1834 bekannt gemachten Verzeichnisse in Wurttemberg vorkommender zweiflugliger Insekten. Correspondenzblatt des Koeniglich Wuerttembergischen Landwirthschaftlichen Vereins, Stuttgart.

[B7081982] Séguy E. (1934). Faune de France 28, Dipteres (Brachyceres: Acalypterae et Scatophagidae).

[B7078244] Seljak G. (2013). The burr-seed fly, *Euarestaaequalis* (Loew) (Diptera: Tephritidae), newly recorded in Europe, with new observations on its biology. Studia Dipterologica.

[B7078253] Shcherbakov M. V. (2001). Three new species of the genus *Tephritis* Latreille (Diptera, Tephritidae) from Southern Siberia. International Journal of Dipterological Researches.

[B7078262] White I. M. (1988). Tephritid flies (Diptera: Tephritidae). Handbooks for Identification of British Insects.

[B7078270] White I. M., Korneyev V. A. (1989). A revision of the western Palaearctic species of *Urophora* Robineau-Desvoidy (Diptera: Tephritidae. Systematic Entomology.

[B7078279] Zarghani E., Khaghaninia S., Mohamadzade Namin S., Karimpour Y., Korneyev V. A. (2016). First records of the fruit flies (Diptera, Tephritidae) in the fauna of Iran. Vestnik Zoologii.

